# Increase in insulin-like growth factor 1 (IGF-1) and insulin-like growth factor binding protein 1 after supplementation with selenium and coenzyme Q10. A prospective randomized double-blind placebo-controlled trial among elderly Swedish citizens

**DOI:** 10.1371/journal.pone.0178614

**Published:** 2017-06-13

**Authors:** Urban Alehagen, Peter Johansson, Jan Aaseth, Jan Alexander, Kerstin Brismar

**Affiliations:** 1 Division of Cardiovascular Medicine, Department of Medical and Health Sciences, Linköping University, Linköping, Sweden; 2 Department of Social and Welfare Studies, Linköping University, Norrköping, Sweden; 3 Research Department, Innlandet Hospital Trust, Brumunddal, Norway, and Hedmark University of Applied Sciences, Elverum, Norway; 4 Norwegian Institute of Public Health, Oslo, and Norwegian University of Life Sciences (NMBU), Ås, Norway; 5 Department of Molecular Medicine and Surgery, Karolinska University Hospital, Karolinska Institutet, Stockholm, Sweden; Garvan Institute of Medical Research, AUSTRALIA

## Abstract

**Background:**

Insulin-like growth factor-1(IGF-1) has a multitude of effects besides cell growth and metabolism. Reports also indicate anti-inflammatory and antioxidative effects. The concentrations of IGF-1 decrease with age and during inflammation. As selenium and coenzyme Q10 are involved in both the antioxidative defense and the inflammatory response, the present study aimed to examine the effects of supplementation with selenium and coenzyme Q10 on concentrations of IGF-1 and its binding protein IGFBP-1 in a population showing reduced cardiovascular mortality following such supplementation.

**Methods:**

215 elderly individuals were included and given the intervention for four years. A clinical examination was performed and blood samples were taken at the start and after 48 months. Evaluations of IGF-1, the age adjusted IGF-1 SD score and IGFBP-1 were performed using group mean values, and repeated measures of variance.

**Findings:**

After supplementation with selenium and coenzyme Q10, applying group mean evaluations, significantly higher IGF-1 and IGF-1 SD scores could be seen in the active treatment group, whereas a decrease in concentration could be seen of the same biomarkers in the placebo group.

Applying the repeated measures of variance evaluations, the same significant increase in concentrations of IGF-1 (F = 68; *P*>0.0001), IGF-1 SD score (F = 29; *P*<0.0001) and of IGFBP-1 (F = 6.88; *P* = 0.009) could be seen, indicating the effect of selenium and coenzyme Q10 also on the expression of IGF-1 as one of the mechanistic effects of the intervention.

**Conclusion:**

Supplementation with selenium and coenzyme Q10 over four years resulted in increased levels of IGF-1 and the postprandial IGFBP-1, and an increase in the age-corrected IGF-1 SD score, compared with placebo. The effects could be part of the mechanistic explanation behind the surprisingly positive clinical effects on cardiovascular morbidity and mortality reported earlier. However, as the effects of IGF-1 are complex, more research on the result of intervention with selenium and coenzyme Q10 is needed.

## Introduction

Circulating insulin-like growth factor-1 (IGF-1) is mainly produced in the liver as a response to stimulus from the growth hormone [1], but is also produced locally in different tissues in the body. It is a 70 amino acid polypeptide with a weight of 7.6 kDa [2], and there are six binding proteins that modulate the effects of IGF-1 [3]. It has anabolic effects in adults, with pleiotropic effects on both cell growth and metabolism. Of the multitude of effects described, it has been reported that overexpression of IGF-1 diminishes myocyte necrosis and apoptosis in myocardial infraction in a mouse model [4]. It has also been demonstrated that there is reduced cardiac myocyte atrophy as a result of ischemia in those with high levels of IGF-1 [5]. IGF-1 also has anti-inflammatory effects and decreases the concentration of pro-inflammatory cytokines [6, 7]. In the normal population there is a well-known decrease of the IGF-1 levels with increasing age [8]. Therefore,

In patients with ischemic heart disease, the amount of coronary calcium deposits has been reported to be inversely associated with the level of IGF-1 [9], and in several studies a low level of IGF-1 has been associated with increased risk of ischemic heart disease or myocardial infarction, as seen in both human and animal models [10–12]. In elderly men, however, Carlzon et al. demonstrated increased risk of cardiovascular events both in those with high and low IGF-1 levels [13].

Scharin Täng et al. reported interesting data from a mouse model indicating that a greater cardiac left ventricular volume and decreased cardiac function could be demonstrated in those with induced absence of IGF-1 [14]. In humans, Andreassen et al. found that low levels of IGF-1 could act as a predictor for heart failure progression and cardiovascular mortality [15]. This has also been demonstrated by others [16, 17].

Thus, there is a clear clinical association between decreased concentration of IGF-1 and disease also in the elderly, as can be seen in the literature [10, 15, 17–21]. As described in the literature, the IGF-binding protein-1 (IGFBP-1) is also associated with cardiovascular disease [17,18]. In previous studies we have shown that supplementation with selenium and coenzyme Q10 in an elderly healthy Swedish population reduced cardiovascular mortality, improved cardiac function [22], and decreased oxidative stress[23] and inflammation [24]. A possible mechanism behind these effects may be an influence on IGF concentrations. As our study population had a mean age of 78 years and the IGF concentration decreases with age [25], we hypothesized that those randomized to selenium and coenzyme Q10 would preserve their high IGF concentration, whereas the placebo group would have a decrease in IGF concentration.

The aim of this sub study was to investigate a possible influence of supplementation with selenium and coenzyme Q10 for four years on IGF-1 and IGFBP-1 in an elderly healthy Swedish population.

## Material and methods

The design of the main study has been published elsewhere [22]. In brief, 443 elderly healthy participants living in a rural municipality in the south of Sweden were randomized to dietary supplementation of 200 mg/day of coenzyme Q10 capsules (Bio-Quinon 100 mg B.I.D, Pharma Nord, Vejle, Denmark) and 200 μg/day of organic selenium yeast tablets (SelenoPrecise 200 μg, Pharma Nord, Vejle, Denmark), or a similar placebo. The study supplementation was taken in addition to regular medication. The participants were examined by one of three experienced cardiologists. A new clinical history was recorded, and a clinical examination was performed, including resting blood pressure, assessment of New York Heart Association functional class (NYHA class) as well as ECG and Doppler- echocardiography. Echocardiographical examinations were performed with the participant in the left lateral position. The ejection fraction (EF) readings were categorized into four classes with interclass limits placed at 30%, 40% and 50% [26, 27]. Normal systolic function was defined as EF≥ 50%, while severely impaired systolic function was defined as EF*<* 30%.

As the intervention time was unusually long (48 months) only 221 participants completed the study, 64 died during the total intervention time, and 129 (29.1%) decided not to complete the study. The reasons for the latter have been presented in detail in the main publication, but the main reason was that there were too many tablets to take [22]. The first participant was included in January 2003, and the last participant concluded the study in February 2010. Out of the total study population, 215 participants were analyzed regarding IGF-1, the age-corrected values of IGF-1 (IGF-1 SD) based on the standard deviation of the mean value based on 247 healthy individuals [28, 29], and the IGF-binding protein 1 (IGFBP-1), which is presented in this report. Of these, 117 had previously been randomized to active treatment (supplementation with selenium and Q10) and 98 to placebo. Thirteen participants missed delivering the final blood samples for the IGF-1 evaluation.

Written, informed consent was obtained from all patients. The study was approved by the Regional Ethical Committee in Linköping, Sweden (Diary no. 03–176), and conforms to the ethical guidelines of the 1975 Declaration of Helsinki. This study was registered at Clinicaltrials.gov, and has the identifier NCT01443780

### Biochemical analyses

All blood samples were obtained while the patients were at rest in a supine position. The blood samples were collected in plastic vials containing EDTA (ethylenediamine tetracetic acid). The vials were placed on ice before chilled centrifugation at 3000g, and then frozen at -70°C. No sample was thawed more than twice.

IGF-1 concentrations in plasma were determined by an in-house RIA after separation of the different IGFs from IGFBPs by acid ethanol extraction and cryoprecipitation. To minimize interference from the remaining IGFBPs, des (1–3) IGF-1 was used as a radioligand [30]. The intra- and inter-assay coefficients of variation (CV) were 4% and 11%, respectively. Plasma concentrations of IGF-1 are age-dependent, decreasing with age, thus IGF-1 values are also expressed as standard scores (SD scores) calculated from the regression of the values of two populations of healthy adult subjects [29, 31–34].

IGFBP-1 concentrations were determined by an in-house RIA using the method of Póvoa et al. [35]. The sensitivity of the RIA was 3 μg/L and the intra- and inter-assays CV were 3% and 10%, respectively.

### Statistical methods

Descriptive data are presented as percentages or mean ± standard deviation (SD). A student’s unpaired two-sided t-test was used for continuous variables and the Chi-square test was used for analysis of one discrete variable. As the dataset demonstrated a slight non-Gaussian distribution, the dataset was transformed in order to obtain a normal distribution, which was controlled through Kolmogorov-Smirnov´s test. Transformed data were used in the t-test evaluations as this evaluation is more sensitive to a non-normal distribution of data. As the ANOVA algorithm can handle a slight non-Gaussian distribution, non-transformed data were applied in the repeated measures of variance evaluation.

Evaluation of the effects of treatment were based both on group mean values, but also on use of a repeated measures of variance analysis where the values of the individual participant were identified during the two different measured time points. *P*-values < 0.05 were considered significant, based on a two-sided evaluation. All data were analyzed using standard software (Statistica v. 13.2, Dell Inc, Tulsa, OK).

## Results

### Study population

**Fig 1:**
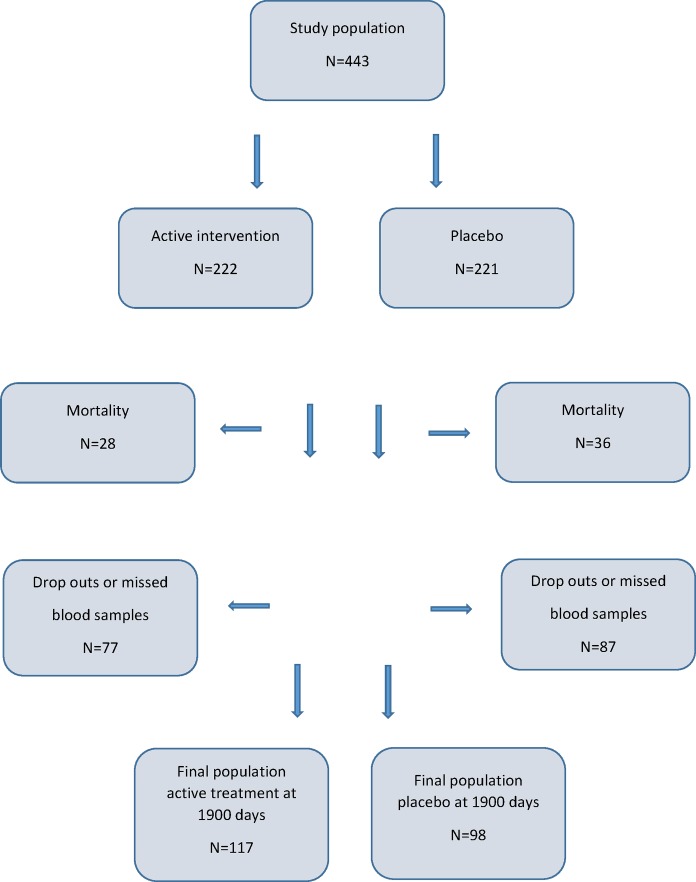
CONSORT flow chart of the study.

The study population of this study consisted of 215 individuals, of which 117 had been given active supplementation consisting of selenium and coenzyme Q10 combined, and 98 individuals had been randomized to placebo (Table 1, Fig 1).

**Table 1:** Basal characteristics of the study population divided into active treatment and placebo, as compared to the total study population.

	Active treatment	P-value	Placebo	Total study population
n	117		98	443
Age years mean (SD)	76.1 (3.1)	0.96	76.2 (3.1)	77.1 (3.5)
History				
Smokers n (%)	8 (6.8)	0.53	9 (7.7)	41 (9.3)
Diabetes n (%)	20 (17.1)	0.96	17 (14.5)	95 (21.4)
Hypertension n (%)	81 (69.2)	0.61	71 (72.4)	326 (73.6)
IHD n (%)	22 (18.8)	0.64	16 (16.3)	100 (22.6)
NYHA class I n (%)	72 (61.5)	0.61	57 (58.2)	226 (51.0)
NYHA class II n (%)	28 (23.9)	0.27	30 (30.6)	125 (28.2)
NYHA class III n (%)	17 (14.5)	0.34	10 (10.2)	88 (19.9)
NYHA class IV n (%)	0		0	0
Unclassified NYHA n (%)	0		1	4 (1.0)
Medications				
ACEI n (%)	16 (13.7)	0.90	14 (14.3)	89 (20.1)
ARB n (%)	3 (2.6)		7 (7.1)	23 (5.2)
Betablockers n (%)	42 (36.0)	0.73	33 (33.7)	153 (34.5)
Digitalis n (%)	5 (4.3)		1 (1.0)	22 (5.0)
Diuretics n (%)	37 (31.6)	0.87	33 (33.7)	158 (35.7)
Statins n (%)	27 (23.1)	0.40	18 (18.4)	96 (21.7)
Examinations				
EF<40% n (%)	7 (6.0)	0.53	4 (4.1)	33 (7.4)

Note: ARB; Angiotension receptor blockers; EF: Ejection fraction; IHD; Ischemic heart disease; NYHA: New York Heart Association functional class

Of the study population, 113 were females, and 102 were males. In the population, about 71% had been diagnosed with hypertension, about 18% had ischemic heart disease, 17% had diabetes, and 5% had an impaired systolic cardiac function defined as EF<40%. The different covariates were well balanced between the active versus the placebo group, and it could also be seen that the subgroup analyzed was representative of the original study population consisting of 443 individuals (Table 1).

At start of the study we were able to demonstrate a trend for higher IGF-1 levels in those already on treatment with ACE-inhibitors, or AII blockers (t = 1.83; *P* = 0.068), a result that could be expected to be significant if a larger study group had been included. This concurs with results from Maggio et al. including elderly patients on treatment with ACE-inhibitors [36].

Evaluations of the IGF-1, IGF-1 SD and IGFBP-1 levels have been performed in relation to basal serum selenium concentration, which varied from 21.4 μg/L Se to 158 μg/L Se, with a median of 67.1 μg/L Se. No significant associations could be found.

### IGF-1 and intervention with selenium and coenzyme Q10

**Fig 2:**
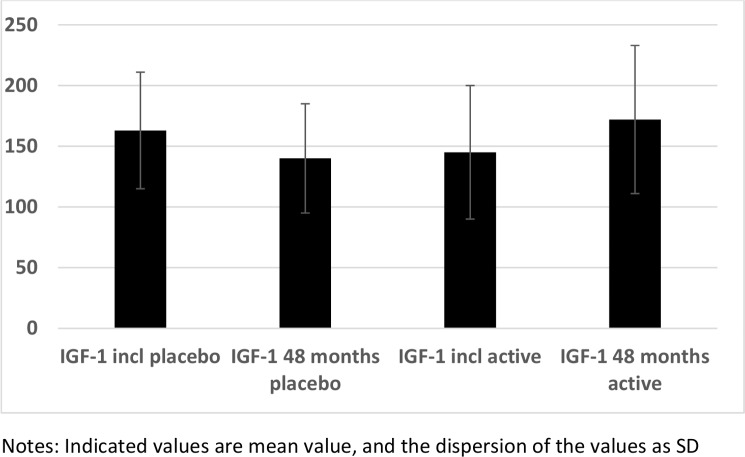
IGF-1 in the active and the placebo groups at the inclusion compared to after 48 months.

At the start of the intervention the serum levels of IGF-1 in the subgroups of those given selenium and coenzyme Q10 combined were not significantly different from values in those given the placebo (t = 1.82; *P* = 0.06) (Fig 2). However, at the end of the intervention period a significantly higher serum level of IGF-1 could be seen in the active treatment group compared with the placebo group (183 vs. 166 microgram/L; t = 5.78; *P*<0.0001). A significant increase in the serum IGF-1 was observed in the active treatment group (from 154 to 183 microgram/L; t = 4.38; *P*<0.0001), whereas a decrease was seen in the placebo group (166 vs. 144 microgram/L; t = 3.43; *P* = 0.0007).

**Fig 3:**
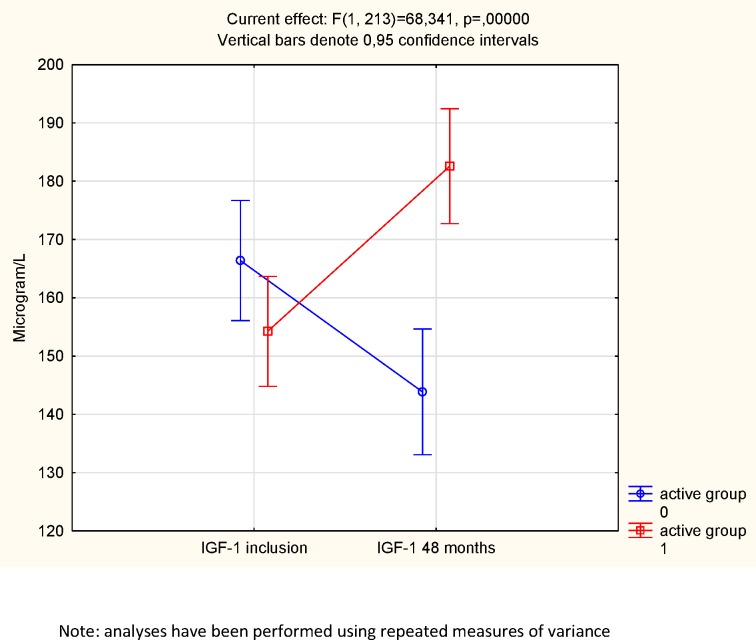
IGF-1 concentration in the active treatment and the placebo groups at the start and at the end of the intervention.

However, as group mean values do not necessarily express the individual change of levels, repeated measures of variance were also performed. Applying repeated measures of variance, using group (active and placebo) and follow-up (baseline and 48 months) a significant effect on serum levels of IGF-1 (i.e. group*follow-up interaction),F = 68; P<0.0001 could be seen, favoring those receiving selenium and coenzyme Q10. Thus, an increase in IGF-1 concentration in those receiving the active substance and a decrease in those given the placebo could be seen (Fig 3.)

**Fig 4:**
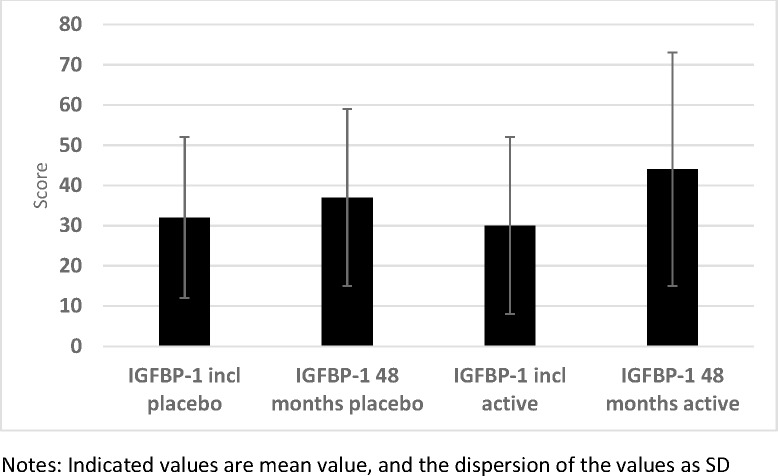
IGF-1 SD score in the active and the placebo groups at inclusion compared to after 48 months.

**Fig 5:**
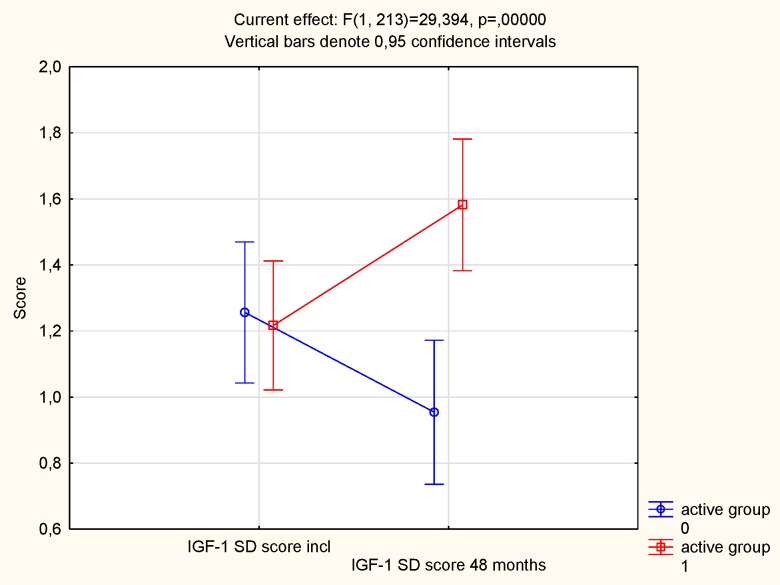
IGF-1 SD score at inclusion on the active treatment and in the placebo groups at the start and at the end of the intervention.

Applying an age-corrected score of IGF-1, the IGF-1 SD, the same result was obtained; at the start of the intervention no statistical difference in score between the active treatment group and the placebo group could be seen (t = 0.06; *P* = 0.96) (Fig 4). However, at the end of the intervention, a highly significant difference could be noted between the groups, with a higher score in the active treatment group (t = 3.11; *P* = 0.002). In the active treatment group, a tendency to increase in score was noted during the intervention (from 1.22 to 1.58; *P* = 0.05), whereas a non-significant decrease was noted in the placebo group (from 1.26 to 0.95; *P* = 0.16). Applying the repeated measure of variance methodology, as reported above, a highly significant effect on IGF-1 SD could be demonstrated (F = 29; P<0.0001)(Fig 5.).

**Fig 6:**
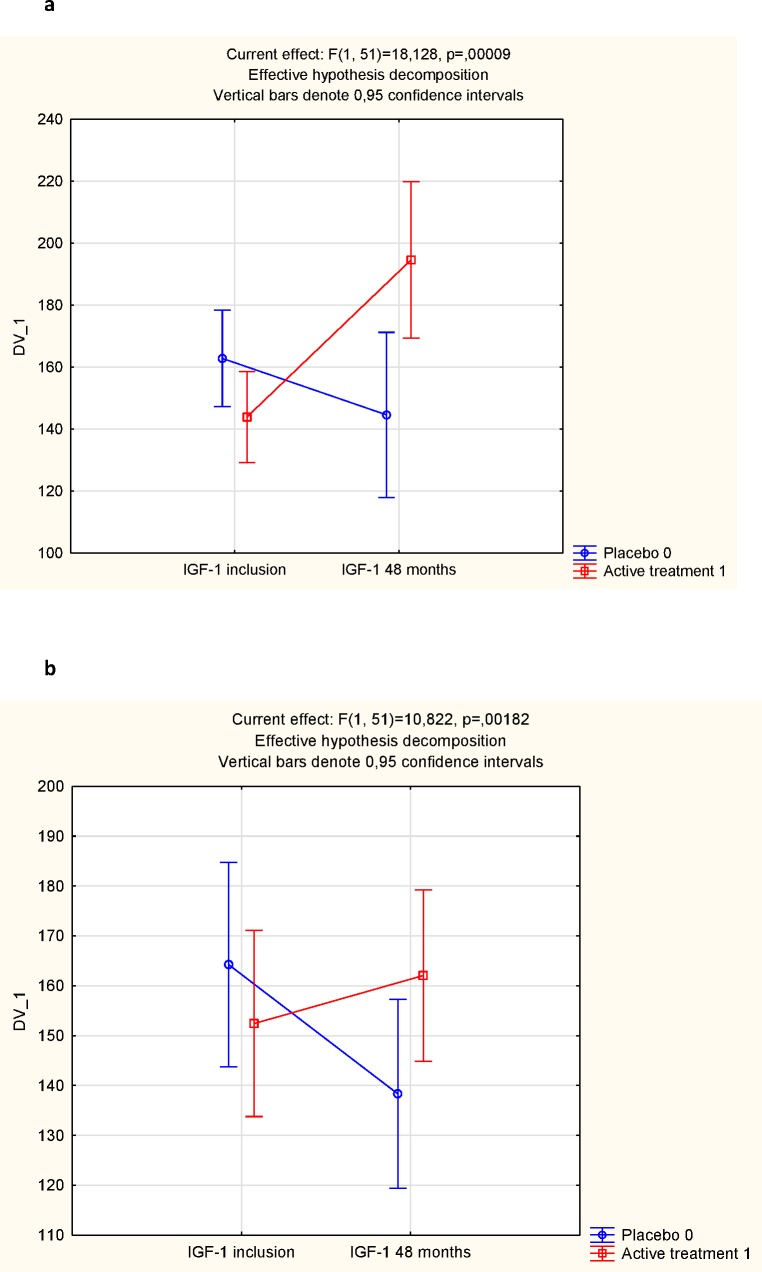
Response to IGF-1 of supplementation with selenium and coenzyme Q10 in those with a basal selenium concentration in the lowest quartile (Fig 6a), and in those with a basal selenium concentration in the highest quartile (Fig 6b).

In order to evaluate the influence of the basal serum selenium concentration on the response to supplementation of selenium and coenzyme Q10 as seen in the concentration of IGF-1, we performed evaluations of the subgroup having the lowest quartile of basal selenium concentration, and compared the response of IGF-1 with those having the highest quartile (Fig 6A and 6B). It could be seen that the response to supplementation with selenium on IGF-1 concentration was significantly higher in those where there was a selenium deficiency compared with those with no, or little deficiency. This is in line with previous reports.

### Estimated active form of IGF-1

In the literature, the use of the ratio of IGF-1/IGFBP-1 as a measure of the active form of IGF-1 has been proposed [37]. A decline in this ratio could be reported in the placebo group during the intervention time (9.72 to 6.31; t = 2.37; *P* = 0.019), whereas in the active treatment group a trend towards a decline during the intervention time could be seen (10.13 to 8.97; t = 2.00; *P* = 0.05), and comparing the ratios of IGF-1/IGFBP-1 between the active and the placebo groups after the intervention a significant difference could be reported (8.97 versus 6.31; t = 2.18; *P* = 0.04).

### IGF-binding protein-1 and intervention with selenium and coenzyme Q10

**Fig 7:**
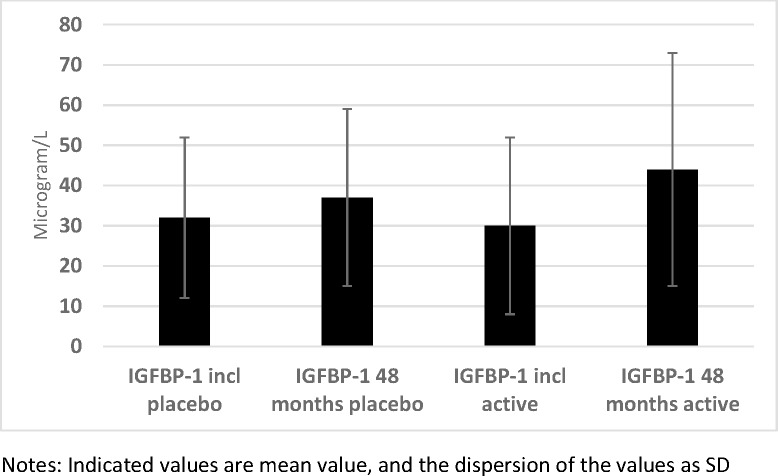
IGFBP-1 in the active and the placebo groups at inclusion compared with after 48 months.

**Fig 8:**
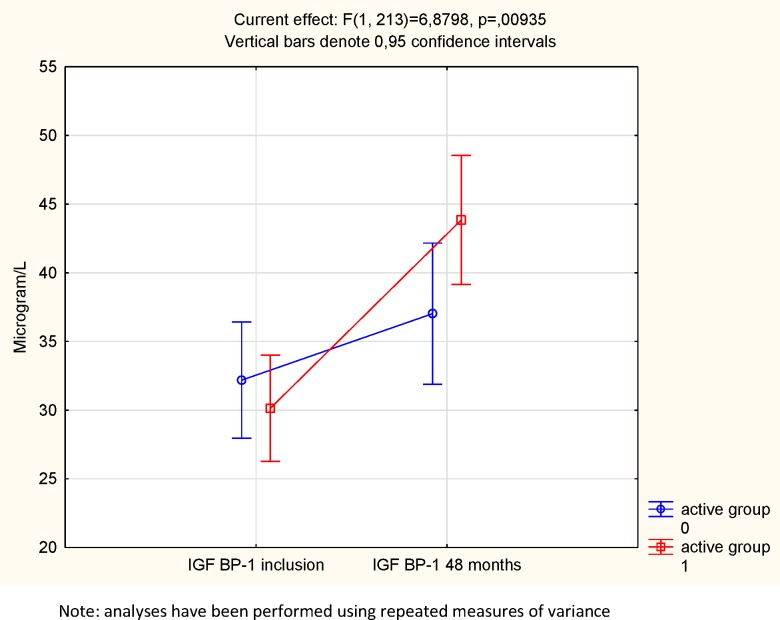
IGFBP-1 concentration in the active treatment and the placebo groups at the start and at the end of the intervention.

The plasma concentrations of one of the six IGF-binding proteins, IGFBP-1, were determined 2–3 hours after a meal. At the start of the intervention, no statistical difference in mean concentrations between the active treatment group, and the placebo group could be noted (30 vs. 32 microgram/L; *P* = 0.25) (Fig 7). At the end of the intervention, no significant difference could be noted (44 vs. 37 microgram/L; *P* = 0.45). However, applying the repeated measure of variance methodology, a significant difference could be demonstrated (F = 6.88; P = 0.009), with a higher increase in the IGFBP-1 concentration in the active group compared with the active treatment group (Fig 8).

We also examined the participants having a concentration of IGFBP-1 in the highest quartile (Q4) and compared the number in the active treatment group with that of the placebo group. No difference in the numbers of participants in Q4 could be seen at the start of the intervention (24/98 versus 29/117: *P* = 0.96). However, after the intervention, compared to the placebo group a significantly higher number of participants could be seen in Q4 of IGFBP-1 from the active treatment group (16/98 versus 36/117; χ^2^ = 6.07; *P* = 0.014).

## Discussion

In the present study we found raised concentrations of IGF-1 and IGF-binding protein-1 in the participants receiving the active intervention, upon the follow-up investigation of the population at the end of the study period. In contrast, we observed decreasing concentrations in those receiving the placebo, which was as expected in this group of aged Swedish individuals [8]. The increase in IGF-1in the supplemented group compared to the placebo group may be connected to the earlier reported decrease in both inflammatory activity and oxidative stress [6] possibly caused by supplemental selenium [38].

As the intervention time in our study was unusually long, four years, and the study population consisted of elderly individuals with a relatively low basal selenium status (41), we hypothesized that there would be a decrease in the levels of IGF-1 in the placebo group and preserved levels in those given active supplementation. In our post hoc analysis, using repeated measurement analyses and an age-corrected dataset, we found raised concentrations of IGF-1 and IGF-binding protein-1 resulting from the active intervention in contrast to the decreasing levels in those receiving the placebo.

In the literature, several pleiotropic effects of IGF-1 have been reported, including antiapoptotic effects [39], and antioxidative effects that could delay atherosclerosis [40, 41]. It has also been shown that IGF-1 increases the circulating number of endothelial progenitor cells that might correct the age-dependent deterioration of the cells [42]. The raised levels of IGF-1 resulting from the intervention, might decrease inflammation and oxidative stress, and promote formation of nitric oxide, and thus relax arterial smooth muscle cells, reduce platelet adhesion and decrease the level of oxidized LDL cholesterol [43–45]. Also, important information was presented by Lohr et al. showing an inverse relationship between IGF-1 and the inflammation biomarker hsCRP [46].

However, upon relating IGF-1, IGF-1 SD and IGFBP-1 levels at baseline to the basal levels of selenium, no significant correlations were seen. Here, it should be noted that the present population was relatively homogenous with respect to selenium status, with median serum selenium of 67.1 μg/L, all individual values being below a proposed optimum of 90 μg/L [47]. In contrast to our finding, Maggio et al. have shown an independent and positive association between selenium levels and IGF-1 in an elderly community-living population comparable to the population examined in the present study, but with a higher selenium status, mean 113 μg/L [48]. By using a mouse model and low doses of selenite, Ren et al. observed both an increased growth and expression of serum IGF-1R and antiapoptotic effects. With higher toxic doses of selenite, growth inhibition, decreased IGF-1R and apoptosis were seen. Similarly, low doses of selenite induced IGF-1R in osteoblasts in vitro whereas with high doses the opposite occurred [49]. Here, the positive effect of selenium might be mediated via an effect on the IGF-1R rather than changes in IGF-1 levels.

When expressing the free and active fraction of IGF-1 as the ratio IGF-1/IGFBP-1, we found that the intervention caused a significantly attenuated decline in the active form of IGF-1. IGFBP-1 was determined after a meal, and we expected its postprandial concentrations to be low due to suppressed expression following the insulin response [33, 50]. The higher IGFBP-1 levels seen in those treated with CoQ10 and selenium suggest a decreased insulin response to the meal due to improved insulin sensitivity. The increased IGF-1 levels induce improved insulin sensitivity in accordance with previous studies showing that antioxidants can improve insulin sensitivity [51, 52]. Our results indicating increased insulin sensitivity are interesting in relation to previous concerns about the possibility that selenium supplementation should cause an increased risk of diabetes [53]. In the present study population, plasma selenium remained well below the upper limit of the considered optimal zone (EFSA, 2014). Furthermore, no increased risk for diabetes (HR:1.09; 95% CI: 0.99–1.20) was reported in one of the biggest meta-analyses (n = 20.294) evaluating the risk for diabetes during selenium supplementation, and also including participants from the US, [54]. Moreover, improved insulin sensitivity may also reduce mortality in old people [55]. This supports the results presented above indicating that selenium and coenzyme Q10 have fundamental effects in the different areas where IGF-1 exerts its actions. In European populations with low basal selenium values our observations suggest that combined supplementation with selenium and coenzyme Q10 may evoke a multitude of effects, including increased IGF-1 and improved insulin sensitivity, which might be among the underlying mechanisms for the positive clinical health effects reported previously.

### Clinical implications

The present subgroup analysis is one of the various efforts to better understand the mechanisms behind the positive clinical results, which include better health-related quality of life [56], better cardiac function[22], and reduced cardiovascular mortality [22] resulting from the intervention with selenium and coenzyme Q10 previously reported. Thus, the analysis provides important data for the clinician, at least in areas with a low basal selenium intake in the general population. Of particular interest here is that the intervention resulted in reduced cardiovascular mortality after 10 years, despite the intervention period lasting only fours [57]. We have also reported less inflammatory activity and less oxidative stress as a result of the intervention, as seen in the biomarkers sP-selectin and hs-CRP [24] and the biomarkers copeptin, and MR-proADM, respectively [23].

It is tempting to argue that in areas with low selenium content in the soil, or where residents have low daily intake of selenium, a moderate supplementation with selenium and coenzyme Q10 will have positive effects, including effects on the levels of IGF-1 in the elderly, as part of the total clinical response, reported above.

## Limitations and strengths

This is a small post hoc study including elderly participants; thus it is not possible at present to extrapolate our results into other age strata. Also, the population was ethnically homogenous, so caution should be used when interpreting the results.

The strength of the report is that the study population was extensively evaluated, and the positive results regarding IGF-1 accord very well with the positive clinical and laboratory results reported earlier.

## Conclusions

IGF-1 has important functions in both cell growth and metabolism, but also in inflammation and antioxidative defense. A decrease in IGF-1 has been reported with increased age, and also in advanced cardiovascular disease.

From our studies on an elderly healthy Swedish population, we have reported in previous publications that more than 90% had suboptimal selenium status [58]. The present evaluation shows that intervention with selenium and coenzyme Q10 resulted in increased levels of IGF-1, as well as in the age-corrected value of IGF-1, and also in the postprandial IGF-binding protein-1 indicative of improved insulin sensitivity. These findings may represent important information on the mechanisms behind the positive clinical effects on cardiovascular morbidity and mortality of supplementation with selenium and coenzyme Q10.

## Supporting information

S1 Study Protocol See appendix. (DOCX)
